# Diagnostic Dilemma, Possible Non-celiac Gluten Sensitivity: Consideration in Approach and Management

**DOI:** 10.7759/cureus.25302

**Published:** 2022-05-24

**Authors:** Uzma Nasim Siddiqui, Aima Pervaiz, Zainab Bashir Khan, Tabassum Sultana

**Affiliations:** 1 General Medicine, Port Macquarie Base Hospital, Port Macquarie, AUS; 2 General Medicine, Shaikh Zayed Federal Postgraduate Medical Institute, Lahore, PAK; 3 Pediatric Medicine, Indus Hospital, Lahore, PAK; 4 Internal Medicine, Shaikh Zayed Federal Postgraduate Medical Institute, Lahore, PAK; 5 Pediatrics and Child Health, Baqai Medical University, Karachi, PAK

**Keywords:** dilemma, approach, possible diagnosis, celiac disease, non celiac gluten sensitivity

## Abstract

Non-celiac gluten sensitivity (NCGS) is clinically identified as a condition where a percentage of the population reports intestinal and/or extraintestinal symptoms caused by gluten and/or wheat ingestion, and they are tested negative for celiac disease (CD) on the basis of specific serology and histopathology. NCGS should be labelled after the exclusion of CD and wheat allergy. This population reports improved symptoms on a gluten-free diet. Despite great interest and work on NCGS, much remains unknown about its pathogenesis. A positive and improved response to a gluten-free diet for a limited period of time (e.g., six to eight weeks), followed by retrieval of symptoms in case of gluten intake, is presently considered to be the best strategy for confirmation of diagnosis.

A middle-aged lady came for medical attention with concerns of weight loss, lethargy and abdominal discomfort. On investigations, her serum transglutaminase IgA was found to be largely raised. The patient was switched to a gluten-free diet with suspicion of CD. Upper GI endoscopy was done one week after being on a gluten-free diet. Both endoscopy with histopathology was negative for villous atrophy and increased intraepithelial lymphocytes. Later human leukocyte antigen (HLA) testing was found to be negative for CD, leading to a diagnostic conundrum. On the basis of remarkable symptom improvement on a gluten-free diet, drop in transglutaminase levels, negative biopsy and HLA testing, the diagnosis was made as possible NCGS. Considering gluten-related disorders are rising and not much is known about NCGS, we aimed to present this case to create awareness and raise questions regarding diagnosis, need for specific monitoring and implications on the management.

## Introduction

Non-celiac gluten sensitivity (NCGS) or commonly known as gluten sensitivity is defined as “a clinical syndrome caused by the gluten ingestion resulting in intestinal and/or extraintestinal symptoms that alleviate once the gluten-containing food is removed from the diet, NCGS is a diagnosis of exclusion after excluding celiac disease and wheat allergy” [1]. Among gluten-related disorders, NCGS is the most recently diagnosed and the most unknown [2], the exact prevalence is yet unknown [3]. There is no biomarker available for the diagnosis. NCGS typically presents with gastrointestinal symptoms similar to celiac disease (CD) and irritable bowel syndrome (IBS) [4]. It is a relatively confusing clinical entity with overlapping symptoms with other gluten-related disorders and with unknown etiology and pathogenesis [5].

## Case presentation

We present a 42-year-old lady who presented with complaints of lethargy, unintentional weight loss of 7 kg in two weeks along with upper abdominal discomfort and bloating. Past medical history was significant for type 2 diabetes mellitus for 10 years with good control on metformin, sitagliptin and basal insulin glargine. She was diagnosed with hypothyroidism and hypertension five years back. Both issues were fairly controlled on thyroxine replacement and amlodipine respectively.

On examination, she was a well-looking lady with a BMI of 24. Her general physical and systemic examination was completely unremarkable. On workup, her baseline investigations were: Hb 10.7 mg/dl; mean corpuscular volume (MCV) 74.5 fl; white blood cells (WBC) 7.7 x 10^9^/L; platelets 249 x 10^9^/L; microcytic, hypochromic, anisocytosis on peripheral smear; erythrocyte sedimentation rate (ESR) 25 mm/h; serum iron 34.0 micrograms/dl; total iron-binding capacity (TIBC) 379 micrograms/dl; transferrin saturation 8.97%; serum ferritin 7.7 ng/ml; negative H. pylori stool antigen; glycosylated hemoglobin (HbA1c) 6.1%; thyroid-stimulating antibodies 0.097 uIU/ml; blood urea nitrogen 11 mg/dl; serum creatinine 0.7 mg/dl; serum electrolytes, liver enzymes, and fasting lipid profile were all in the normal ranges; 25-hydroxy vitamin D 19.5; stool and urine microscopy was not significant.

**Figure 1:**
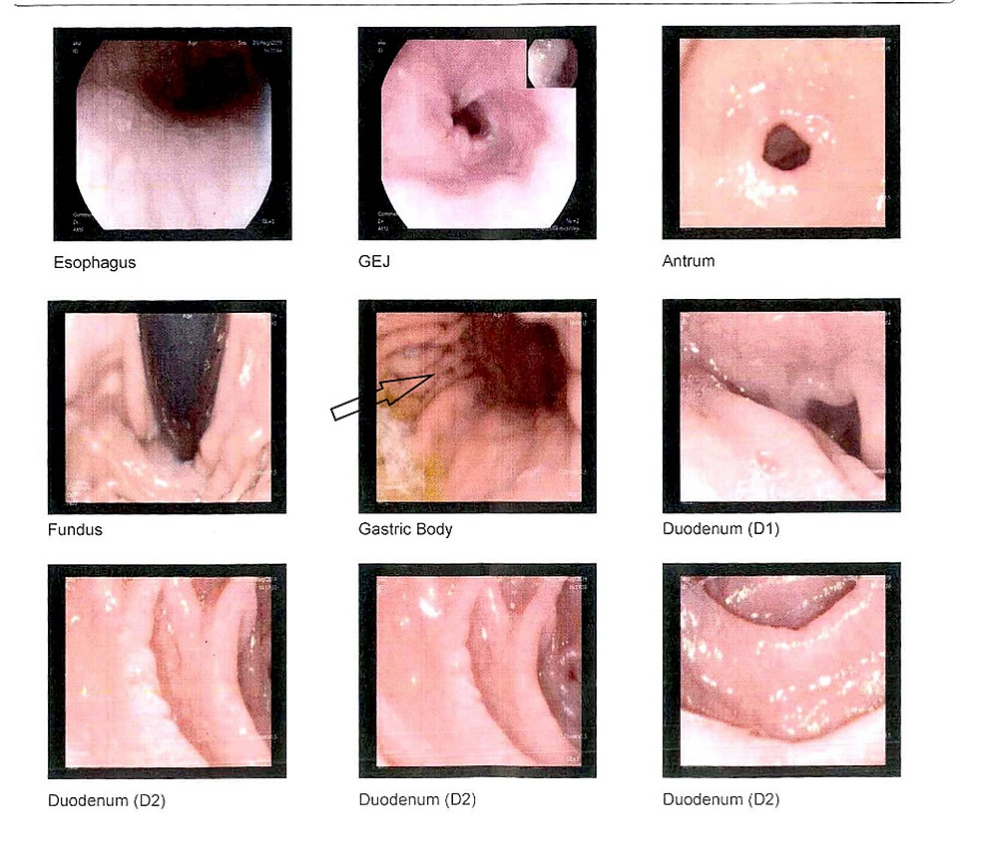
Endoscopic views of the esophagus, stomach and duodenum showing normal esophagus, mild erythema in the gastric body, antrum and mucosal fissuring in the first and distal part of the duodenum. GEJ: Gastroesophageal junction

**Figure 2:**
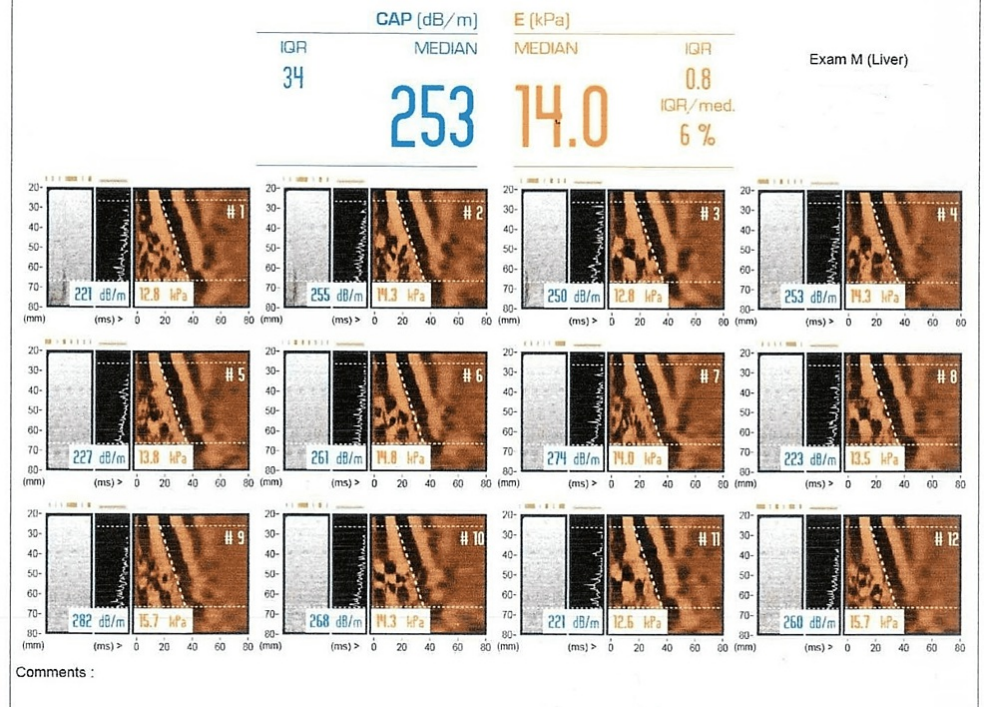
Fibroscan showing grade 1 steatosis with grade 4 fibrosis

The patient was managed with hematinics, vitamin D and calcium supplementations. On the follow-up visit after two weeks, no significant difference in terms of symptoms was observed. Further panel of tests were carried out including serum cortisol morning 14.8 mcg/dL, serum B12 192 pg/ml, serum transglutaminase IgA >100, and serum IgA 4.4. With the suspicion of CD, the patient was put on a gluten-free diet, and to further strengthen the diagnosis, the patient was planned for a diagnostic workup. Upper gastrointestinal endoscopy was performed one week after being on a gluten-free diet. Six biopsy specimens were taken from the antrum, duodenum and duodenal bulb. Biopsy revealed fragments of duodenal mucosa with preserved villoglandular architecture and moderate nonspecific duodenitis and focal mild chronic active gastritis (Figure 1). No features of intestinal metaplasia or malignancy were observed. The patient’s abdominal scan showed mild coarse and echogenic liver parenchyma representing early chronic inflammatory changes with mild to moderate fatty infiltration but regular margins. Portal vein was found to be prominent measuring 13.8 mm. In view of ultrasound findings, further workup was done. Autoimmune profile antinuclear antibody (ANA), anti-smooth muscle antibody (ASMA), double-stranded DNA (dsDNA), serum liver kidney microsome (LKM), and serum ceruloplasmin levels were all in the normal ranges. Hepatitis B surface antigen (HBsAg) and anti-hepatitis C virus (HCV) antibody was nonreactive (Table 1). Fibro scan showed grade 1 steatosis with grade 4 fibrosis (Figure 2).

**Table 1:** List of investigations MCV: Mean corpuscular volume; WBC: White blood cells; ESR: Erythrocyte sedimentation rate; BUN: Blood urea nitrogen; CRP: C-reactive protein; TSH: Thyroid-stimulating hormone; HDL: High-density lipoprotein; LDL: Low-density lipoprotein; VLDL: Very low-density lipoprotein; Gamma GT: Gamma-glutamyl transferase; ALT: Alanine aminotransferase; AST: Aspartate aminotransferase; PT: Prothrombin time; INR: International normalized ratio; WBC/HPF: White blood cells per high-powered field; HBsAg: Hepatitis B surface antigen; HCV: Hepatitis C virus; LKM: Liver kidney microsome; ANA: Antinuclear antibody; HLA: Human leukocyte antigen; AMA: Antimitochondrial antibody.

Hb 10.7 mg/dl	CRP 1.34
MCV 74.5 fL	Albumin 4.4 g/dl
WBC 7.7 x 10^9^/L	PT 10.1
Platelets 249 x 10^9^/L	INR 0.9
ESR 25 mm/hour	Glucose random 99 mg/dl
Serum iron 34.0 microgram/dl	Serum 25-hydroxy vitamin D 19.5 ng/ml
Total iron-binding capacity (TIBC) 379 micrograms/dl	Serum calcium 8.9 mg/dl
Transferrin saturation 8.97%	Serum B12 192 pg/ml
Serum ferritin 7.1 ng/ml	Stool analysis. occasional WBC/HPF
Glycosylated hemoglobin (HbA1c) 6.1%	H. pylori stool antigen Negative
TSH 0.097 uIU/ml	Serum cortisol 14.80 ug/dl
BUN 11 mg/dl	Serum anti-transglutaminase IgA >100 U/ml
Serum cholesterol 98 mg/dl	Serum anti-transglutaminase IgG <0.5
Serum triglycerides 110 mg/dl	HBsAg Non-reactive
HDL cholesterol 54 mg/dl	HCV antibody non-reactive
LDL cholesterol 54 mg/dl	Serum LKM IgG 1.14 U/ML
VLDL cholesterol 22 mg/dl	Serum ceruloplasmin 0.28 G/L
Total bilirubin 0.5 mg/dl	Anti-DsDNA 2.01 IU/ml
Direct bilirubin 0.2 mg/dl	AMA negative
Indirect bilirubin 0.3 mg/dl	ANA negative
Gamma GT 45 IU/L	HLA DQ2 Negative
ALT 70 IU/L	HLA DQ8 Negative
Alk Phosphatase 79 IU/L	
AST 64 IU/L	
Serum Na 137 mmol/L	
Serum potassium 4.1 mmol/L	

As the upper gastrointestinal endoscopy and histopathology were not consistent with the presumptive diagnosis of CD, HLA DQ2 and DQ8 genotype testing was done and was found to be negative. On reevaluation of the patient four weeks after being on a gluten-free diet, significant improvement in symptoms along with 2 kg weight gain was noted. Follow-up labs revealed a rise in Hb from 10.7 to 12.5 g/dl, and a decline in levels of serum anti-transglutaminase IgA from >100 to <0.5 U/ml. Serum anti-transglutaminase IgG remained static with <0.5U/ml (Table 2).

**Table 2:** List of investigations after gluten restriction MCV: Mean corpuscular volume

Serum anti-transglutaminase IgA <0.5 U/ml
Serum anti-transglutaminase IgG <0.5 U/ml
Hb 12.5 g/dl
MCV 81.5 fL
WBC 9.0 x 10^9^/L
Platelets 177 x 10^9^/L

In view of remarkable improvement in symptoms and fall of anti-transglutaminase levels on a gluten-free diet and absence of histopathological evidence and HLA testing, the final diagnosis was made as possible non-celiac gluten sensitivity and liver cirrhosis due to non-alcoholic steatohepatitis (NASH) (based on fibro scan results) secondary to diabetes mellitus and hypothyroidism.

She was advised to keep long-life dietary restrictions for gluten and follow up as directed.

## Discussion

Gluten-related disorders comprise CD, wheat allergy and non-celiac gluten sensitivity. Initially, the gluten-related disorder was recognized as CD only, later on, with increased work in this field different entities were described [6]. In this case, there was no evidence of wheat allergy but the patient was suspected to have CD as the patient’s anti-tissue transglutaminase (anti-tTG) antibodies were high along with intestinal manifestations (abdominal bloating and weight loss). Significant improvement in symptoms with weight gain following a gluten-free diet was observed. The gold standard test for CD, i.e. the small bowel biopsy, was negative for specific histological features (villous atrophy and increased intraepithelial lymphocytes), and to our surprise, celiac-associated DQ markers, HLQ DQ2 and DQ8, were also negative. Keeping in view the negative predictive value of HLQ haplotypes (99%), CD was excluded [7].

Resolution of symptoms on a gluten-free diet and restoration of symptoms on the reintroduction of gluten leads to the diagnosis of NCGS [8-9]. Antigliadin antibodies are usually found in more than half of the patients with gluten sensitivity with the absence of specific markers of celiac disease [10]. In the index patient’s serum anti-transglutaminase IgA levels were reduced to <0.5 from >100 after excluding gluten from the diet and intestinal symptoms were improved. The presence of serum anti-transglutaminase IgA before the exclusion of gluten from the diet is against the diagnosis of NCGS [10] but there is no other alternative explanation which could be attributed. Although tissue transglutaminase IgA is quite specific for the diagnosis of CD but solely should not be used for the establishment of diagnosis. It is worth mentioning that upper GI endoscopic view in the index patient, some mucosal fissuring was noted in the antrum and duodenum without scalloping or villous atrophy. These findings are not specific to CD and are especially not useful in the presence of dyspeptic symptoms [11]. In certain cases, the biopsy can be false negative where the biopsy specimen is not enough or if the biopsy is not taken from the duodenal bulb [12]. Furthermore, negative histopathology is also possible when the patient had been on gluten restriction, but complete histopathological recovery can take around two years [13]. These considerations were especially taken into account before reporting this case and also endoscopy was done only after a week, being on a gluten-free diet. Therefore, the chances of having CD are remote in this case. In case of negative histopathology on a gluten-free diet, HLA testing is usually advised. The chances of having CD can be ruled out in the absence of HLA haplotypes [12-13]. Apart from intestinal, a number of extra-intestinal manifestations (headache, chronic fatigue and depression) could be associated with NCGS. They were not seen in this patient although autoimmunity underlying hypothyroidism and diabetes mellitus of this patient could be reportedly associated with NCGS. However, positive HLA typing (DQ2) has been seen in NCGS [14]. The case is quite a diagnostic dilemma. Hence, the question arises that despite negative histopathology HLA typing and positive serology, gluten-related symptoms could be attributed to NCGS. Although perhaps treatment would not differ much. The NCGS diagnosis is often challenging due to the overlap of symptoms with other gluten-related disorders and nonexistence of any specific biomarkers. For future perspective, there is a need for more research and development of a diagnostic arsenal in this area [15].

## Conclusions

Diagnosis of NCGS is often a challenge due to the lack of specific diagnostic criteria, specific biomarkers and the presence of overlapping symptoms. There is a need of future research in this area as existing medical literature also cannot answer the need of any required monitoring in NCGS.
